# Changes in the rankings of leading causes of death in Japan, Korea, and Taiwan from 1998 to 2018: a comparison of three ranking lists

**DOI:** 10.1186/s12889-022-13278-7

**Published:** 2022-05-10

**Authors:** Shu-Yu Tai, Soyeon Cheon, Yui Yamaoka, Yu-Wen Chien, Tsung-Hsueh Lu

**Affiliations:** 1 Department of Family Medicine, School of Medicine, College of Medicine, Kaohsiung Municipal Ta-Tung Hospital and Kaohsiung Medical University Hospital, Kaohsiung Medical University, Kaohsiung, Taiwan; 2 Department of Public Health, College of Medicine, National Cheng Kung University, Tainan, Taiwan; 3 Department of Global Health Promotion, Tokyo Medical and Dental University, Tokyo, Japan

**Keywords:** International Classification of Disease, International comparisons of mortality, Japan, Korea, Leading causes of death, Rankings, Taiwan

## Abstract

**Background:**

The ranking lists used by most countries for leading causes of death (CODs) comprise broad category such as cancer, heart disease, and accidents. To provide more specific information, the World Health Organization (WHO) and the Institute of Health Metrics and Evaluation (IHME) proposed lists that splitting broad categories into specific categories. We examined the changes in rankings of leading CODs according to different lists in Japan, Korea, and Taiwan from 1998 to 2018.

**Methods:**

We obtained the number of deaths for three countries from the WHO mortality database for 1998, 2008, and 2018. Age-standardized death rates were calculated for rankings 10 leading CODs using WHO 2000 age structure as standard.

**Results:**

The first leading COD was cancer in Japan, Korea, and Taiwan from 1998 to 2018 based on government list; nevertheless, became stroke based on WHO list, and was stroke and ischemic heart disease based on IHME list. In the WHO and IHME lists, cancer is categorized based on cancer site. The number of cancer sites included in the 10 leading CODs in 2018 was 4, 4, and 3 in Japan, Korea, and Taiwan, respectively according to the WHO list and was 4, 4, and 2, respectively according to IHME list. The only difference was the rank of liver cancer in Taiwan, which was 6th according to WHO list and was 18th according to IHME list. The ranking and number of deaths for some CODs differed greatly between the WHO and IHME lists due to the reallocation of “garbage codes” into relevant specific COD in IHME list.

**Conclusions:**

Through the use of WHO and the IHME lists, the relative importance of several specific and avoidable causes could be revealed in 10 leading CODs, which could not be discerned if the government lists were used. The information is more relevant for health policy decision making.

**Supplementary Information:**

The online version contains supplementary material available at 10.1186/s12889-022-13278-7.

## Background

The rankings of leading causes of death (CODs) are highly cited health statistics by mass media and health-related advocacy organizations to illustrate the relative importance of particular health issues. Many countries publish official rankings of 10 leading CODs every year [1–5]. However, the ranking lists used by these countries comprise broad category such as cancer, heart disease, and accidents. The category of cancer composes 89 three-character *International Classification of Disease Tenth Revision* (*ICD-10*) codes (*C00-C97*) for different cancer sites. Accidents (unintentional injuries) category composes more than 100 three-character *ICD-10* codes (*V01-X59*). On the contrary, only 1 three-character *ICD-10* codes (*G30*) is included in category of Alzheimer’s disease. It is an unfair comparison between ranking categories [6].

Furthermore, from the disease prevention point of view, cancers of different sites are heterogeneous in terms of etiology, progression, screening capability, and treatment modalities. Grouping these cancer sites with various levels of preventability and treatability into one category for mortality rankings cannot provide specific and actionable information. Similarly, injury control strategies for road traffic injuries differ from those for unintentional poisoning or falls. Grouping various unintentional injuries into one category could not provide specific information to determine the relative importance of health problems [6].

To provide more specific information for health policy decision making and planning, the World Health Organization (WHO) and the Institute of Health Metrics and Evaluation (IHME) proposed that the broad categories in the ranking list should be divided into more specific categories. Table 1 illustrates number of categories and differences in *ICD-10* codes included in selected categories in ranking lists used by government of different countries, the WHO and the IHME. Studies have demonstrated different profiles of rankings of leading CODs between those used government list and those used the WHO list [6–9]. However, little is known whether the profiles of rankings according to the IHME list differed from previous lists as the IHME list reallocated “garbage codes” into specific CODs categories. Therefore, we aimed in this study to examine the changes in rankings of leading CODs in Japan, Korea, and Taiwan (three countries located in the West Pacific region) from 1998 to 2018 based on different ranking lists, namely government lists, the WHO list, and the IHME list.

**Table 1:** Differences in ICD-10 codes included in selected ranking categories in lists used by government of Japan, Korea, Taiwan, the United States (US), the World Health Organization (WHO) and the Institute of Health Metrics and Evaluation (IHME)

	Japan	Korea	Taiwan	US	WHO	IHME
No. of categories	39	56	40	52	65	137
**Broad categories in government lists but are split in the WHO and the IHME lists**						
Cancer	C00-C97	C00-C97	C00-C97	C00-C97	17 categories	29 categories
Heart disease	I01-I02.0, I05-I09, I20-I25, I27, I30-I52	I20-I51	I01-I02.0, I05-I09, I20-I25, I27, I30-I52	I00-I09, I11, I13, I20-I51	8 categories	10 categories
Accidents	V01-X59	5 categories	V01-X59, Y85-Y86	V01-X59, Y85-Y86	6 categories	13 categories
**Some categories combined**						
Dementia	F01-F03	NA	F01-F03	NA	F01,F03,G30	F00-F03.9, G30-G31.1, G31.8-G31.9
Alzheimer disease	G30	G30	G30	G30		
Influenza	J10-J11	J09-J11	J10-J11	J10-J18	J10-J18	A48.1, A70, B97.4-B97.6, J09-J15.8, J16-J16.9, J20-J21.9, U04-U04.9
Chronic lower respiratory diseases	J41-J44	J40-J47	J40-J47	J40-J47	J40-J47	J41-J44.9
	J45-J46					J45-J46.9
**Similar category name, but are slightly different in ICD-10 codes included**						
Diabetes mellitus	E10-E14	E10-E14	E10-E14	E10-E14	E10-E14	E10-E10.1, E10.3-E11.1, E11.3-E11.9
Parkinson disease	G20	NA	G20-G21	G20-G21	G20	G20-G20.9
Hypertension	I10-I15	I10-I13	I10-I15	I10, I12	I10-I15	I11-I11.9
Cirrhosis and other diseases of liver	K70-K77	K70-K76	K70, K73-K74	K70, K73-K74	K70-K76	B18-B18.9, I85-I85.9, I98.2, K70-K70.3, K71.7, K74-K74.9, K75.2, K75.4-K76.2, K76.4-K76.9, K77.8

The complete categories in each ranking list was illustrated in Supplementary Table S1, S2, S3, S4, S5 and S6

## Methods

We obtained the number of deaths for various ranking categories (Supplementary Table S1,S2, S3 and S5 to S6) for Japan and Korea from the WHO mortality database for 1998, 2008, and 2018. As Taiwan is not the member country of the WHO, the number of deaths for ranking categories for Taiwan were retrieved from open data released by the Ministry of Health and Welfare, Taiwan [10]. For the 10 leading CODs according to the IHME list, we used the “GBD Results Tool” to retrieve the number of deaths and percentages for the 10 leading CODs in each country over the study period [11].

Since the age structure differed across countries and years, the age-standardized death rates were calculated for rankings of 10 leading CODs using WHO 2000 age structure as standard. This study was approved by the Institute of Review Board of the National Cheng Kung University Hospital (B-EX-110–018).

## Results

The number of deaths in Japan, Korea, and Taiwan was 936,484, 245,825, and 121,946 respectively in 1998 and increased to 1,362,470, 298,820, and 172,859, respectively in 2018. The first leading COD was cancer in Japan, Korea, and Taiwan from 1998 to 2018 based on government list (Tables 2 , 3 and 4). However, according to WHO list, the first leading COD was stroke for 1998, 2008, and 2018 in Japan (Table 2) and Korea (Table 3); stroke for 1998 and 2008 and influenza and pneumonia for 2018 in Taiwan (Table 4). Based on IHME list, the first leading COD was stroke for 1998 and 2008 and ischemic heart disease for 2018 in Japan (Table 2); stroke for all study years in Korea (Table 3); stroke for 1998 and ischemic heart disease for 2008 and 2018 in Taiwan (Table 4).

**Table 2:** Rankings of 10 leading causes of death based on three ranking lists in Japan, 1998, 2008, and 2018

	1998 (*N* = 936,484)			2008 (*N* = 1,142,407)			2018 (*N* = 1,362,470)		
Rank	Cause	%	Rate	Cause	%	Rate	Cause	%	Rate
	Government list								
1	Cancer	30.3	226.7	Cancer	30.0	272.3	Cancer	27.4	300.7
2	Heart Dis	15.3	114.3	Heart Dis	15.9	144.4	Heart Dis	15.3	167.6
3	Stroke	14.7	110.0	Stroke	11.1	100.9	Senility	8.0	88.2
4	Pneumonia	8.5	63.8	Pneumonia	10.1	91.6	Stroke	7.9	87.1
5	Accidents	4.2	31.1	Accidents	3.3	30.3	Pneumonia	6.9	76.2
6	Suicide	3.4	25.4	Senility	3.1	28.6	Accidents	3.0	33.2
7	Senility	2.3	17.1	Suicide	2.6	24.0	Asp Pneu	2.8	31.0
8	Renal failure	1.8	13.3	Renal failure	2.0	17.9	Renal failure	1.9	21.0
9	Liver Dis	1.7	12.9	Liver Dis	1.4	12.9	Dementia	1.5	16.5
10	Diabetes	1.3	10.0	COPD	1.4	12.3	Suicide	1.5	16.1
	World Health Organization list								
1	Stroke	14.7	56.3	Stroke	11.1	36.6	Stroke	7.9	24.5
2	Influ & Pneu	8.6	31.6	Influ & Pneu	10.1	29.3	Influ & Pneu	7.2	18.5
3	IHD	7.7	29.8	IHD	6.7	23.4	Lung ca	5.5	18.3
4	Stomach ca	5.4	22.6	Lung ca	5.9	21.6	IHD	5.1	17.0
5	Lung ca	5.4	22.0	Suicide	2.8	20.2	Heart failure	6.4	16.9
6	Suicide	3.5	20.4	Heart failure	5.7	17.6	Suicide	1.7	14.3
7	Heart failure	4.8	18.7	Stomach ca	4.4	16.6	Colon ca	3.8	13.6
8	Colon ca	3.7	15.4	Colon ca	3.8	14.5	Stomach ca	3.2	11.1
9	Liver ca	3.6	15.1	Liver ca	2.9	11.2	Pancreas ca	2.6	9.3
10	Transport Inj	1.4	8.5	Pancreas ca	2.3	8.7	Urinary Dis	2.9	7.6
	Institute of Health Metrics and Evaluation list								
1	Stroke	13.6	60.3	Stroke	11.0	38.8	IHD	10.0	29.9
2	IHD	12.0	52.9	IHD	10.7	38.1	Stroke	10.2	29.6
3	Influ & Pneu	8.1	36.2	Demen & AD	9.0	28.3	Demen & AD	11.8	27.7
4	Stomach Ca	5.9	25.6	Influ & Pneu	8.3	27.7	Influ & Pneu	8.4	22.2
5	Lung Ca	6.0	25.4	Lung Ca	6.5	24.1	Lung Ca	6.2	21.2
6	Demen & AD	5.4	24.4	Suicide	2.8	18.7	Colon Ca	4.6	15.8
7	Colon Ca	4.2	18.6	Stomach Ca	4.9	18.7	Suicide	1.8	14.3
8	Suicide	3.2	17.5	Colon Ca	4.4	16.9	Stomach Ca	4.1	14.1
9	Liver Ca	3.7	16.1	Liver Ca	3.1	11.9	Pancreas Ca	2.7	9.6
10	Kidney Dis	2.8	12.5	Kidney Dis	3.1	10.6	Kidney Dis	3.2	8.9

*Ca* Cancer, *Cirrhosis* Liver cirrhosis, *CLRD* Chronic Lower Respiratory Disease, *Demen & AD* Dementia & Alzheimer Disease, *Diabetes* Diabetes Mellitus, *Dis* Disease, *IHD* Ischemic Heart Disease, *Influ & Pneu* Influenza & Pneumonia, *Inj* Injuries

The rate was age-standardized using WHO 2000 age structure as standard

**Table 3:** Rankings of 10 leading causes of death based on three ranking lists in Korea, 1998, 2008, and 2018

	1998 (*N* = 245,825)			2008 (*N* = 246,113)			2018 (*N* = 298,820)		
Rank	Cause	%	Rate	Cause	%	Rate	Cause	%	Rate
	Government list								
1	Cancer	20.9	109.5	Cancer	28.0	139.5	Cancer	26.5	154.3
2	Stroke	14.1	74.1	Stroke	11.3	56.5	Heart Dis	10.7	62.4
3	Heart Dis	7.4	38.9	Heart Dis	8.7	43.4	Pneumonia	7.8	45.4
4	Transport inj	4.9	25.8	Suicide	5.2	26.0	Stroke	7.7	44.7
5	Liver Dis	4.7	24.8	Diabetes	4.2	20.7	Suicide	4.6	26.6
6	Diabetes	4.0	21.2	CLRD	3.0	14.9	Diabetes	2.9	17.1
7	Suicide	3.5	18.6	Transport inj	3.0	14.7	Liver Dis	2.3	13.4
8	CLRD	2.4	12.7	Liver Dis	2.9	14.5	CLRD	2.2	12.9
9	Hypertension	1.6	8.5	Pneumonia	2.2	11.1	Alzheim Dis	2.1	12.0
10	Resp TB	1.4	7.2	Hypertension	1.9	9.6	Hypertension	2.0	11.8
	World Health Organization list								
1	Stroke	14.1	93.1	Stroke	11.3	47.9	Stroke	7.7	23.9
2	Stomach ca	4.6	28.2	Suicide	6.3	27.1	Influ & Pneu	8.0	23.6
3	Cirrhosis	4.7	26.8	Lung ca	6.0	24.7	Suicide	5.1	22.3
4	Transport Inj	4.9	26.1	IHD	5.2	21.7	Lung ca	6.0	19.3
5	Diabetes	4.0	25.9	Liver ca	4.6	18.5	IHD	4.9	15.2
6	Lung ca	3.9	24.8	Diabetes	4.2	17.3	Liver ca	3.6	12.1
7	Liver ca	3.8	22.7	Stomach ca	4.2	17.3	Colon ca	2.9	9.5
8	Suicide	3.9	20.1	CLRD	3.0	12.8	Demen & AD	3.3	9.2
9	IHD	3.1	19.8	Transport Inj	2.9	12.7	Diabetes	2.9	9.2
10	CLRD	2.4	16.6	Cirrhosis	2.9	11.7	Stomach ca	2.6	8.6
	Institute of Health Metrics and Evaluation list								
1	Stroke	18.1	145.8	Stroke	13.3	70.1	Stroke	11.3	43.8
2	IHD	8.5	72.5	IHD	8.6	47.4	IHD	8.8	34.8
3	Stomach Ca	6.5	42.1	Lung Ca	6.6	29.0	Lung Ca	7.3	26.1
4	Cirrhosis	6.0	35.6	Demen & AD	4.1	26.7	Demen & AD	5.8	24.2
5	Lung Ca	5.3	35.1	Suicide	6.1	26.0	Influ & Pneu	5.0	20.2
6	Transport Inj	5.7	30.1	Diabetes	5.0	23.6	Suicide	4.8	19.7
7	Diabetes	4.2	28.2	Stomach Ca	5.1	22.5	Liver Ca	4.6	16.1
8	Asthma	2.7	27.4	Liver Ca	4.9	20.5	Diabetes	4.0	15.0
9	Demen & AD	2.2	26.7	COPD	3.2	18.2	Stomach Ca	3.8	14.0
10	COPD	2.5	23.4	Influ & Pneu	2.7	16.3	Colon Ca	3.7	13.7

*Ca* Cancer, *Cirrhosis* Liver cirrhosis, *CLRD* Chronic Lower Respiratory Disease, *Demen & AD* Dementia & Alzheimer Disease, *Diabetes* Diabetes Mellitus, *Dis* Disease, *IHD* Ischemic Heart Disease, *Influ & Pneu* Influenza & Pneumonia, *Inj* Injuries

The rate was age-standardized using WHO 2000 age structure as standard

**Table 4:** Rankings of 10 leading causes of death based on three ranking lists in Taiwan, 1998, 2008, and 2018

	1998 (*N* = 121,946)			2008 (*N* = 142,283)			2018 (*N* = 172,859)		
Rank	Cause	%	Rate	Cause	%	Rate	Cause	%	Rate
	Government list								
1	Cancer	24.0	134.0	Cancer	27.3	133.7	Cancer	28.2	121.8
2	Stroke	10.4	58.2	Heart Dis	11.1	51.7	Heart Dis	12.5	48.8
3	Heart Dis	9.0	50.5	Stroke	7.5	35.0	Pneumonia	7.8	27.4
4	Accidents	9.0	50.3	Pneumonia	6.1	27.5	Stroke	6.7	26.1
5	Diabetes	6.2	34.5	Accidents	5.0	27.0	Diabetes	5.4	21.5
6	Cirrhosis	4.1	22.6	Diabetes	5.6	26.9	Accidents	4.0	21.1
7	Pneumonia	3.6	20.4	Cirrhosis	3.5	17.1	Hypertension	3.5	12.8
8	Kidney Dis	2.8	15.7	CLRD	3.8	16.9	CLRD	3.6	12.7
9	Hypertension	1.9	10.4	Suicide	2.9	15.2	Suicide	2.2	12.5
10	Suicide	1.8	10.0	Kidney Dis	2.8	13.2	Kidney Dis	3.2	12.3
	World Health Organization list								
1	Stroke	10.4	61.5	Stroke	7.5	34.8	Influ & Pneu	7.9	31.7
2	Diabetes	6.2	36.2	Influ & Pneu	6.1	27.3	Stroke	6.7	27.9
3	Liver ca	4.8	27.8	Diabetes	5.6	26.6	IHD	5.9	24.7
4	Lung ca	4.7	27.4	Liver ca	5.4	26.3	Lung ca	5.4	23.6
5	Transport Inj	4.9	27.0	Lung ca	5.5	26.0	Diabetes	5.4	22.7
6	IHD	4.5	26.8	IHD	5.5	25.7	Liver ca	4.8	20.9
7	Cirrhosis	4.6	26.0	Urinary Dis	4.3	19.9	Urinary Dis	5.1	20.7
8	CLRD	4.1	24.3	Cirrhosis	3.9	19.3	Heart failure	4.7	19.9
9	Influ & Pneu	3.6	21.9	Suicide	3.3	17.1	Colon ca	3.4	14.6
10	Urinary Dis	3.6	21.6	CLRD	3.8	16.9	Hypertension	3.5	14.1
	Institute of Health Metrics and Evaluation list								
1	Stroke	12.3	82.8	IHD	9.2	49.0	IHD	9.1	41.8
2	IHD	8.9	64.6	Stroke	9.0	47.1	Stroke	8.0	37.5
3	Diabetes	7.3	46.3	Diabetes	6.5	33.7	Influ & Pneu	7.1	32.8
4	Cirrhosis	5.8	34.4	Influ & Pneu	6.0	33.0	Lung Ca	6.5	30.4
5	Lung Ca	5.3	32.1	Lung Ca	6.4	32.7	Diabetes	6.0	28.1
6	COPD	3.9	29.8	Colon Ca	4.7	24.4	Colon Ca	5.6	26.2
7	Influ & Pneu	3.9	29.3	Cirrhosis	4.8	23.8	COPD	4.6	20.9
8	Transport Inj	5.3	28.6	COPD	4.2	23.1	Demen & AD	4.7	20.9
9	Kidney Dis	3.2	22.3	Demen & AD	3.7	21.0	Kidney Dis	4.4	20.4
10	Colon Ca	3.3	20.9	Kidney Dis	3.8	20.2	Cirrhosis	3.9	19.2

*Ca* Cancer; Cirrhosis: Liver cirrhosis, *CLRD* Chronic Lower Respiratory Disease, *Demen & AD* Dementia & Alzheimer Disease, *Diabetes* Diabetes Mellitus, *Dis* Disease, *IHD* Ischemic Heart Disease, *Influ & Pneu* Influenza & Pneumonia, *Inj* Injuries

The rate was age-standardized using WHO 2000 age structure as standard

In the WHO and IHME lists, cancer is categorized based on cancer site. The number of specific cancer sites included in the 10 leading CODs in 2018 was 4 (lung followed by colon, stomach, and pancreas) in Japan (Table 2), 4 (lung followed by liver, colon, and stomach) in Korea (Table 3), and 3 (lung followed by liver and colon) in Taiwan (Table 4), respectively according to the WHO list and was 4, 4, and 2, respectively according to IHME list. The only difference was the rank of liver cancer in Taiwan, which was 6th according to WHO list and was 18th according to IHME list.

We noted considerable variation in rankings and percentages of some CODs between those according to the WHO list and those according to the IHME list. For dementia and Alzheimer’s disease in 2018, the proportion of deaths according to the WHO list was lower than those according to the IHME list, which was 2.9% vs. 11.8%, respectively in Japan (Table 2), 3.3% vs. 5.8%, respectively in Korea (Table 3), and 0.9% vs. 4.7%, respectively in Taiwan (Table 4). For liver cancer in 2018, the proportion was 1.9% vs. 2.5%, respectively in Japan and 3.6% vs. 4.6%, respectively in Korea. On the contrary, the proportion of deaths according to the WHO list was larger than those according to the IHME list in Taiwan, which was 4.8% vs. 1.5%, respectively.

## Discussion

### Main findings

The findings of this study reveal different profiles of rankings of the 10 leading CODs between Japan, Korea, and Taiwan across years based on different ranking lists. Through the use of the WHO and the IHME lists, the relative importance of several specific and avoidable causes (lung cancer, colon cancer, liver cancer, ischemic heart disease, transportation injuries) could be revealed in 10 leading CODs, which could not be discerned if government lists were used. We also noted that the ranks and number of deaths for some CODs differed greatly between those according to the WHO and those according to the IHME list due to the reallocation of “garbage codes” into relevant specific COD in IHME list.

### In relation to previous studies

Several studies also illustrated different rankings of leading CODs according to government list and the WHO list [6–9]. Becker et al. used ranking list splitting cancer into specific cancer sites for mortality data in western Europe in 2001 revealed the top four leading CODs as ischemic heart disease, lung cancer, stroke, and heart failure in contrast to cancer, ischemic heart disease, stroke and heart failure according to ranking list without splitting cancer [7]. Griffiths et al. applied the ranking list splitting both cancer and accidents to 2003 mortality data in England and Wales illustrated that the first and second leading CODs was ischemic heart disease and stroke, respectively for both men and women. However, for the third and fourth leading CODs, it was lung cancer and chromic lower respiratory diseases, respectively for men and was influenza and pneumonia and dementia and Alzheimer’s disease, respectively for women [8]. Hsiao et al. used the same ranking list for mortality data for years 2008–2012 in Taiwan and demonstrated different profile of rankings of 10 leading CODs in Taiwan. The top four leading CODs according government list was cancer, heart disease, stroke, and pneumonia and was stroke, influenza and pneumonia, diabetes mellitus, and lung cancer according to the WHO list [9]. A recent study indicated that COVID-19 was the third leading COD in the United States in 2020 according to the NCHS ranking list and was the second leading COD according to the WHO ranking list (the number of death for COVID-19 was 345,323 instead of 435,323 in that study, a typo error) [6].

A recent US study compared the top 10 leading CODs between 2011 and 2018 noted that accidents ranked the fifth followed by cancer, heart disease, chronic lower respiratory diseases, stroke in 2011 and increased to the third followed by cancer and heart disease in 2018. The increase of ranking of accidents highlights the emergent public health problem and mainly due to the increase of drug overdose [12]. That study also indicated that number of deaths in each leading COD increased from 2011 to 2018; nevertheless, the age-adjusted death rates decreased in seven CODs due to the aging of population and increased in three CODs (accidents, Alzheimer’s disease, and suicide) [12].

### The need of using specific categories for ranking

One key feature of the WHO and IHME lists was the division of the broad cancer category into subcategories based on cancer sites. One important evidence to support the division is that not all cancer sites are avoidable CODs. According to the November 2019 version of OECD/Eurostat lists of preventable and treatable causes of death, only sixteen cancers are included in the avoidable mortality indicators. Nine cancers (oral, esophageal, stomach, liver, lung, mesothelioma, skin [melanoma], bladder, and cervical cancer) are classified as preventable through the implementation of effective public health and primary prevention interventions and eight cancers (cervical, colorectal, female breast, uterus, testicular, thyroid cancer, Hodgkin disease, and lymphoid leukemia) are classified as treatable (or amenable) through timely and effective health care interventions [13].

Through this division, we could identify which cancer sites were ranked higher as CODs in the countries. For example, according to the WHO list, stomach cancer and liver cancer were ranked relatively high in Japan and Taiwan, respectively. Furthermore, through comparisons of the rankings of CODs across different countries across years, we could identify which cancer site was an emergent health concern in a given country, such as with colon cancer in Japan and Taiwan. Through the use of the WHO list, we could get more specific and actionable information with regard to the relative importance of cancers of various sites.

The second broad category—heart disease—has been divided into 9 categories in the WHO list and 10 categories in the IHME level 3 list. Nevertheless, many of the subcategories for heart disease in the WHO list are mechanisms of death (been referred to “garbage codes” by GBD Study group) such as cardiac arrest, cardiac arrhythmias, and heart failure, which could not provide specific information for disease prevention and control. Heart failure ranked high in Japan (fifth in 2003 and 2008 and increased to the third in 2013 and 2018) than those in Korea (not within 10 leading CODs) and Taiwan (tenth in 2008, ninth in 2013, and eighth in 2018). This is possibly associated with the different death certification behaviors across countries.

The third broad category—accidents—has been divided into 6 categories in the WHO list and 13 categories in the IHME level 3 list. The government ranking list in Korea has divided accidents into specific unintentional injury subcategories. According to government list in Japan, accidents ranked the fifth in 1998, 2003, and 2008 and the sixth in 2013 and 2018; in Taiwan, accidents ranked the fourth in 1998, the fifth in 2003, the sixth in 2008, 2013, and 2018. However, based on the WHO list, no unintentional injury subcategory was within the 10 leading CODs in Japan and only transportation injury ranked the fifth in 1998 and the eighth in 2003 in Taiwan.

### Differences between the WHO and IHME lists

We noted sizable differences in the rankings and percentages of deaths for some CODs between the WHO and IHME lists. The first explanation is the differences in *ICD-10* codes included in ranking categories. For example, the *ICD-10* codes for dementia and Alzheimer’s disease are F01, F03, and G30 in the WHO list (Table 1) and F00-F02.0, F02.8-F03.91, F06.2, G30-G31.1, and G31.8-G32.89 in the IHME list (Table 1). Another example is the inclusion of 6 three-character *ICD-10* codes (I10-I15) for hypertensive diseases in the WHO list; yet, only one three-character *ICD-10* codes (I11) for hypertensive heart disease in the IHME list.

A second explanation is the reallocation of “garbage codes” to relevant specific codes in the GBD Study, which was used in the IHME lists [14–16]. In 2015, the top three “garbage codes” were heart failure (4.6%), senility (4.4%), and pneumonitis (1.7%) in Japan; senility (4.5%), ill-defined (3.2%), and heart failure (1.7%) in Korea; and unspecified primary or secondary liver cancer without specified (4.3%), sepsis (2.1%), and heart failure (2.1%) in Taiwan. The number of deaths from dementia and Alzheimer’s disease in Japan in 2015 before and after allocation of garbage codes was 25,109 and 141,017, respectively, which is a 5.6-fold difference mainly due to a high proportion of deaths from senility being reallocated as dementia and Alzheimer’s disease deaths. Similarly, in Taiwan, many of the deaths due to liver cancer reported by the WHO list have been reallocated as secondary liver cancer, which decreased the number of deaths on the IHME list [16].

### Limitations

Regarding the limitations of this study, caution should be applied when interpreting rankings of the leading CODs. First, physicians in different countries might have different preferences in using different diagnoses for the same disease. For example, Japanese physicians were more likely to use “heart failure” and “senility” diagnoses than physicians in Korea and Taiwan. Furthermore, Korean physicians were more likely to use “Alzheimer disease” than their counterpart physicians in Japan and Taiwan, who prefer to use “dementia.” Second, because of too many stratifications (by 3 years and by three ranking lists and by three countries), we did not further calculate rankings of CODs by age and sex in this study. Third, the details of reallocation of “garbage codes” into specific CODs by IHME were not available. Further studies on some CODs with large differences (dementia and Alzheimer’s disease in Japan and liver cancer in Taiwan) were needed.

## Conclusions

This study examined changes in the rankings of 10 leading CODs from 1998 to 2018 in Japan, Korea, and Taiwan by using three ranking lists (government, WHO, and IHME lists). Dividing the broad categories in government lists into more specific COD categories, as the WHO and IHME lists have done, can provide more specific and actionable information for health policy decision makers. Furthermore, harmonization and clarification to improve the comparability of different ranking lists is required to facilitate interpretation of rankings of CODs between countries and across years. Finally, as emphasized by Becker et al., an analysis of the rankings of leading CODs is only a starting point for overall analysis of the mortality profile of a country, and in most cases, further supplemental analyses should be performed [7].

## Supplementary Information

**Additional file 1. Table S1. **List for ranking leading causes of death by government of Japan

**Additional file 2. Table S2. **List for ranking leading causes of death by government of Korea

**Additional file 3. Table S3. **List for ranking leading causes of death by government of Taiwan

**Additional file 4.** **Table S4. **List for ranking leading causes of death by government of the United States

**Additional file 5. Table S5. **List for ranking leading causes of death by the World Health Organization

**Additional file 6. Table S6. **List for ranking leading causes of death by the Institute of Health Metrics and Evaluation

## Data Availability

The datasets used and/or analyzed during the current study are available from the corresponding author on reasonable request.

## Abbreviations

Ca: Cancer
Cirrhosis: Liver cirrhosis
CLRD: Chronic Lower Respiratory Disease
Demen & AD: Dementia & Alzheimer Disease
Diabetes: Diabetes Mellitus
Dis: Disease
ICD-10: International Classification of Disease Tenth Revision
IHD: Ischemic Heart Disease; IHME: Institute of Health Metrics and Evaluation
Influ & Pneu: Influenza & Pneumonia
Inj: Injuries
NCHS: National Center for Health Statistics
WHO: World Health Organization
