# Supplementary material for: Changes in the rankings of leading causes of death in Japan, Korea, and Taiwan from 1998 to 2018: a comparison of three ranking lists
Source: BMC Public Health. 2022 May 10;22:926. doi: 10.1186/s12889-022-13278-7 (PMC9086411; doi:10.1186/s12889-022-13278-7)
Supplement: Supplementary file 1 — Additional file 1. Table S1. List for ranking leading causes of death by government of Japan [file 12889_2022_13278_MOESM1_ESM.docx]

Table S1. List for ranking leading causes of death by government of Japan (N=39)

| **Number** | **Name of category** | **ICD-10 codes** |
| --- | --- | --- |
| 1100 | Intestinal infectious diseases | A00-A09 |
| 1200 | Tuberculosis | A15-A19 |
| 1300 | Septicemia | A40-A41 |
| 1400 | Viral hepatitis | B15-B19 |
| 1500 | Human immunodeficiency virus (HIV) disease | B20-B24 |
| 2100 | Malignant neoplasms | C00-C97 |
| 2200 | In situ neoplasms, benign neoplasms and neoplasms of uncertain or unknown behavior | D00-D48 |
| 3100 | Anemias | D50-D64 |
| 4100 | Diabetes mellitus | E10-E14 |
| 5100 | Vascular and unspecified dementia | F01-F03 |
| 6100 | Meningitis | G00-G03 |
| 6200 | Spinal muscular atrophy and related syndromes | G12 |
| 6300 | Parkinson disease | G20 |
| 6400 | Alzheimer disease | G30 |
| 9100 | Hypertensive diseases | I10-I15 |
| 9200 | Diseases of heart（except hypertensive diseases） | I01-I02.0, I05-I09,I20-I25, I27, I30-I52 |
| 9300 | Cerebrovascular diseases | I60-I69 |
| 9400 | Aortic aneurysm and dissection | I71 |
| 10100 | Influenza | J10-J11 |
| 10200 | Pneumonia | J12-J18 |
| 10300 | Acute bronchitis | J20 |
| 10400 | Chronic obstructive pulmonary disease | J41-J44 |
| 10500 | Asthma | J45-J46 |
| 11100 | Peptic ulcer | K25-K27 |
| 11200 | Hernia | K40-K46, K56 |
| 11300 | Diseases of liver | K70-K77 |
| 12000 | Diseases of the skin and subcutaneous tissue | L00-L99 |
| 13000 | Diseases of the musculoskeletal system and connective tissue | M00-M99 |
| 14100 | Glomerular and renal tubulo-interstitial diseases | N00-N16 |
| 14200 | Renal failure | N17-N19 |
| 15000 | Pregnancy, childbirth and the puerperium | O00-O99 |
| 16000 | Certain conditions originating in the perinatal period | P00-P96 |
| 17000 | Congenital malformations, deformations and chromosomal abnormalities | Q00-Q99 |
| 18100 | Senility | R54 |
| 18200 | Sudden infant death syndrome（SIDS） | R95 |
| 20100 | Accidents (unintentional injuries) | V01-X59 |
| 20200 | Intentional self-harm (suicide) | X60-X84 |
| 20300 | Assault (homicide) | X85-Y09 |
| 22100 | Severe acute respiratory syndrome [SARS] | U04 |
