# Supplementary material for: Changes in the rankings of leading causes of death in Japan, Korea, and Taiwan from 1998 to 2018: a comparison of three ranking lists
Source: BMC Public Health. 2022 May 10;22:926. doi: 10.1186/s12889-022-13278-7 (PMC9086411; doi:10.1186/s12889-022-13278-7)
Supplement: Supplementary file 2 — Additional file 2. Table S2. List for ranking leading causes of death by government of Korea [file 12889_2022_13278_MOESM2_ESM.docx]

Table S2. List for ranking leading causes of death by government of Korea

| **Number** | **Name of category** | **ICD-10 codes** |
| --- | --- | --- |
| 1 | Cholera | A00 |
| 2 | Other gastoenteritis and colitis of infectious and unspecified origin | A09 |
| 3 | Other intestinal infectious disease | A01-A08 |
| 4 | Respiratory tuberculosis | A15-A16 |
| 5 | Other tuberculosis | A17-A19 |
| 6 | Plague | A20 |
| 7 | Tetanus | A33-A35 |
| 8 | Diphtheria | A36 |
| 9 | Whooping cough | A37 |
| 10 | Meningococcal infection | A39 |
| 11 | Septicaemia | A40-A41 |
| 12 | Infections with a predominantly sexual mode of transmission | A50-A64 |
| 13 | Acute poliomyelitis | A80 |
| 14 | Rabies | A82 |
| 15 | Yellow fever | A95 |
| 16 | Other arthropod-borne viral fevers and viral haemorrhagic fevers | A90-A94, A96-A99 |
| 17 | Measles | B05 |
| 18 | Viral hepatitis | B15-B19 |
| 19 | Human immunodeficiency virus[HIV] disease | B20-B24 |
| 20 | Malaria | B50-B54 |
| 21 | Leishmaniasis | B55 |
| 22 | Trypanosomiasis | B56-B57 |
| 23 | Schistosomiasis | B65 |
| 24 | Remainder of certain infectious and parasitic diseases | A21-A32,A38,A42-A49,A65-A79,A81,A83-A89,B00-B04,B06-B09,B25-B49,B58-B64,B66-B94,B99,U07.1, U07.2 |
| 25 | Malignant neoplasms | C00-C97 |
| 26 | Anaemias | D50-D64 |
| 27 | Diabetes mellitus | E10-E14 |
| 28 | Malnutrition | E40-E46 |
| 29 | Mental and behavioural disorders due to psychoactive substance use | F10-F19 |
| 30 | Meningitis | G00,G03 |
| 31 | Alzheimer's disease | G30 |
| 32 | Acute rheumatic fever and chronic rheumatic heart diseases | I00-I09 |
| 33 | Hypertensive diseases | I10-I13 |
| 34 | Heart diseases | I20-I51 |
| 35 | Cerebrovascular diseases | I60-I69 |
| 36 | Atherosclerosis | I70 |
| 37 | Influenza | J09-J11 |
| 38 | Pneumonia | J12-J18 |
| 39 | Other acute lower respiratory infections | J20-J22, U04 |
| 40 | Chronic lower respiratory diseases | J40-J47 |
| 41 | Gastric and duodenal ulcer | K25-K27 |
| 42 | Diseases of liver | K70-K76 |
| 43 | Glomerular and renal tubulo-interstitial diseases | N00-N15 |
| 44 | Pregnancy with abortive outcome | O00-O07 |
| 45 | Other direct obstetric deaths | O10-O92 |
| 46 | Indirect obstetric deaths | O98-O99 |
| 47 | Certain conditions originating in the perinatal period | P00-P96 |
| 48 | Congenital malformations, deformations and chromosomal abnormalities | Q00-Q99 |
| 49 | Sudden infant death syndrome | R95 |
| 50 | Transport accidents | V01-V99 |
| 51 | Falls | W00-W19 |
| 52 | Accidental drowning and submersion | W65-W74 |
| 53 | Exposure to smoke, fire and flames | X00-X09 |
| 54 | Accidental poisoning by and exposure to noxious substances | X40-X49 |
| 55 | Intentional self-harm | X60-X84 |
| 56 | Assault | X85-Y09 |
