# Supplementary material for: Changes in the rankings of leading causes of death in Japan, Korea, and Taiwan from 1998 to 2018: a comparison of three ranking lists
Source: BMC Public Health. 2022 May 10;22:926. doi: 10.1186/s12889-022-13278-7 (PMC9086411; doi:10.1186/s12889-022-13278-7)
Supplement: Supplementary file 3 — Additional file 3. Table S3. List for ranking leading causes of death by government of Taiwan [file 12889_2022_13278_MOESM3_ESM.docx]

Table S3. List for ranking leading causes of death by government of Taiwan (N=40)

| **Number** | **Name of category** | **ICD-10 codes** |
| --- | --- | --- |
| 01 | Intestinal infectious diseases | A00-A09 |
| 02 | Tuberculosis | A15-A19 |
| 03 | Septicemia | A40-A41 |
| 04 | Viral hepatitis | B15-B19 |
| 05 | Human immunodeficiency virus (HIV) disease | B20-B24 |
| 06 | Malignant neoplasms | C00-C97 |
| 07 | In situ neoplasms, benign neoplasms and neoplasms of uncertain or unknown behavior | D00-D48 |
| 08 | Anemias | D50-D64 |
| 09 | Diabetes mellitus | E10-E14 |
| 10 | Vascular and unspecified dementia | F01-F03 |
| 11 | Meningitis | G00, G03 |
| 12 | Spinal muscular atrophy and related syndromes | G12 |
| 13 | Parkinson disease | G20-G21 |
| 14 | Alzheimer disease | G30 |
| 15 | Hypertensive diseases | I10-I15 |
| 16 | Diseases of heart（except hypertensive diseases） | I01-I02.0, I05-I09, I20-I25, I27, I30-I52 |
| 17 | Cerebrovascular diseases | I60-I69 |
| 18 | Atherosclerosis | I70 |
| 19 | Aortic aneurysm and dissection | I71 |
| 20 | Influenza | J10-J11 |
| 21 | pneumonia | J12-J18 |
| 22 | Acute bronchitis and bronchiolitis | J20-J21 |
| 23 | Chronic lower respiratory diseases | J40-J47 |
| 24 | Pneumoconiosis | J60-J65 |
| 25 | Pneumonitis due to solids and liquids（except pneumoconiosis） | J66, J68-J69 |
| 26 | Peptic ulcer | K25-K28 |
| 27 | Hernia | K40-K46, K56 |
| 28 | Chronic liver disease and cirrhosis | K70, K73-K74 |
| 29 | Cholelithiasis and other disorders of gallbladder | K80-K82 |
| 30 | Diseases of the skin and subcutaneous tissue | L00-L99 |
| 31 | Diseases of the musculoskeletal system and connective tissue | M00-M99 |
| 32 | Nephritis, nephrotic syndrome and nephrosis | N00-N07, N17-N19, N25-N27 |
| 33 | Pregnancy, childbirth and the puerperium | O00-O99 |
| 34 | Certain conditions originating in the perinatal period | P00-P96 |
| 35 | Congenital malformations, deformations and chromosomal abnormalities | Q00-Q99 |
| 36 | Senility | R54 |
| 37 | Sudden infant death syndrome（SIDS） | R95 |
| 38 | Accidents (unintentional injuries) | V01-X59, Y85-Y86 |
| 39 | Intentional self-harm (suicide) | X60-X84, Y87.0 |
| 40 | Assault (homicide) | X85-Y09, Y87.1 |
