# Supplementary material for: Changes in the rankings of leading causes of death in Japan, Korea, and Taiwan from 1998 to 2018: a comparison of three ranking lists
Source: BMC Public Health. 2022 May 10;22:926. doi: 10.1186/s12889-022-13278-7 (PMC9086411; doi:10.1186/s12889-022-13278-7)
Supplement: Supplementary file 4 — Additional file 4. Table S4. List for ranking leading causes of death by government of the United States [file 12889_2022_13278_MOESM4_ESM.docx]

Table S4. List for ranking leading causes of death by government of the United States (N=50)

| **Number** | **Name of category** | **ICD-10 codes** |
| --- | --- | --- |
| 1# | Salmonella infections | A01-A02 |
| 2# | Shigellosis and amebiasis | A03, A06 |
| # | Tuberculosis | A16-A19 |
| 6# | Whooping cough | A37 |
| 7# | Scarlet fever and erysipelas | A38, A46 |
| 8# | Meningococcal infection | A39 |
| 9# | Septicemia | A40-A41 |
| 10# | Syphilis | A50-A53 |
| 11# | Acute poliomyelitis | A80 |
| 12# | Arthropod-borne viral encephalitis | A83-A84, A85.2 |
| 13# | Measles | B05 |
| 14# | Viral hepatitis | B15-B19 |
| 15# | Human immunodeficiency virus (HIV) disease | B20-B24 |
| 16# | Malaria | B50-B54 |
| # | Malignant neoplasms | C00-C97 |
| 41# | In situ neoplasms, benign neoplasms and neoplasms of uncertain or unknown behavior | D00-D48 |
| 42# | Anemias | D50-D64 |
| 43# | Diabetes mellitus | E10-E14 |
| # | Nutritional deficiencies | E40-E64 |
| 46# | Meningitis | G00,G03 |
| 47# | Parkinson's disease | G20-G21 |
| 48# | Alzheimer's disease | G30 |
| # | Diseases of heart | I00-I09, I11, I13, I20-I51 |
| 60# | Essential (primary) hypertension and hypertensive renal disease | I10, I12 |
| 61# | Cerebrovascular diseases | I60-I69 |
| 62# | Atherosclerosis | I70 |
| 63# | Aortic aneurysm and dissection | I71 |
| # | Influenza and pneumonia | J10-J18 |
| 68# | Acute bronchitis and bronchiolitis | J20-J21 |
| # | Chronic lower respiratory diseases | J40-J47 |
| 74# | Pneumoconioses and chemical effects | J60-J66, J68 |
| 75# | Pneumonitis due to solids and liquids | J69 |
| 77# | Peptic ulcer | K25-K28 |
| 78# | Diseases of appendix | K35-K38 |
| 79# | Hernia | K40-K46 |
| # | Chronic liver disease and cirrhosis | K70, K73-K74 |
| 82# | Cholelithiasis and other disorders of gallbladder | K80-K82 |
| # | Nephritis, nephrotic syndrome and nephrosis | N00-N07, N17-N19, N25-N27 |
| 87# | Infections of kidney | N10-N12, N13.6, N15.1 |
| 88# | Hyperplasia of prostate | N40 |
| 89# | Inflammatory diseases of female pelvic organs | N70-N76 |
| # | Pregnancy, childbirth and the puerperium | O00-O99 |
| 92# | Certain conditions originating in the perinatal period | P00-P96 |
| 93# | Congenital malformations, deformations and chromosomal abnormalities | Q00-Q99 |
| # | Accidents (unintentional injuries) | V01-X59, Y85-Y86 |
| # | Intentional self-harm (suicide) | X60-X84, Y87.0 |
| # | Assault (homicide) | X85-Y09, Y87.1 |
| 109# | Legal intervention | Y35, Y89.0 |
| 112# | Operations of war and their sequelae | Y36, Y89.1 |
| 113# | Complications of medical and surgical care | Y40-Y84, Y88 |
| # | Enterocolitis due to Clostridium difficile | A04.7 |
| # | COVID-19 | U07.1 |
