# Supplementary material for: Changes in the rankings of leading causes of death in Japan, Korea, and Taiwan from 1998 to 2018: a comparison of three ranking lists
Source: BMC Public Health. 2022 May 10;22:926. doi: 10.1186/s12889-022-13278-7 (PMC9086411; doi:10.1186/s12889-022-13278-7)
Supplement: Supplementary file 5 — Additional file 5. Table S5. List for ranking leading causes of death by the World Health Organization [file 12889_2022_13278_MOESM5_ESM.docx]

Table S5. List for ranking leading causes of death by the World Health Organization (N=65)

| **Number** | **Name of category** | **ICD-10 codes** |
| --- | --- | --- |
| LC-01 | Intestinal infectious diseases | A00-A09 |
| LC-02 | Tuberculosis | A15-A19 |
| LC-03 | Vector-borne diseases and rabies | A20, A44, A75-A79, A82-A84, A852, A90-A96, A98.0-A98.2, A98.8, B50-B57 |
| LC-04 | Vaccine-preventable diseases | A33-A37, A80, B01, B05, B06, B15, B16, B17.0, B18.0, B18.1, B18.9, B19, B26 |
| LC-05 | Meningitis | A39, A87, G00-G03 |
| LC-06 | Septicaemia | A40-A41 |
| LC-07 | Human immunodeficiency virus [HIV] disease | B20-B24 |
| LC-08 | Malignant neoplasm of esophagus | C15 |
| LC-09 | Malignant neoplasm of stomach | C16 |
| LC-10 | Malignant neoplasm of colon, sigmoid, rectum and anus | C18-C21 |
| LC-11 | Malignant neoplasm of liver and intrahepatic bile ducts | C22 |
| LC-12 | Malignant neoplasm of gallbladder and other parts of biliary tract | C23, C24 |
| LC-13 | Malignant neoplasm of pancreas | C25 |
| LC-14 | Malignant neoplasm of larynx | C32 |
| LC-15 | Malignant neoplasm of trachea, bronchus and lung | C33, C34 |
| LC-16 | Melanoma and other malignant neoplasms of skin | C43, C44 |
| LC-17 | Malignant neoplasms of female breast | C50 |
| LC-18 | Malignant neoplasm of uterus | C53-C55 |
| LC-19 | Malignant neoplasm of ovary | C56 |
| LC-20 | Malignant neoplasm of prostate | C61 |
| LC-21 | Malignant neoplasm of kidney, except renal pelvis | C64 |
| LC-22 | Malignant neoplasm of bladder | C67 |
| LC-23 | Malignant neoplasm of brain | C71 |
| LC-24 | Malignant neoplasms of lymphoid, haematopoietic and related tissue | C81-C96 |
| LC-25 | Benign neoplasms, in situ and uncertain behavior | D00-D48 |
| LC-26 | Diabetes | E10-E14 |
| LC-27 | Malnutrition and nutritional anemias | D50-D53, E40-E64 |
| LC-28 | Disorders of fluid, electrolyte and acid-based balance dehydration | E86-E87 |
| LC-29 | Dementia and Alzheimer disease | F01, F03, G30 |
| LC-30 | Mental and behavioral disorders due to psychoactive substance use | F10-F19 |
| LC-31 | Parkinson’s disease | G20 |
| LC-32 | Epilepsy and status epilepticus | G40, G41 |
| LC-33 | Chronic rheumatic heart diseases | I05-I09 |
| LC-34 | Hypertensive diseases | I10-I15 |
| LC-35 | Ischemic heart diseases | I20-I25 |
| LC-36 | Pulmonary heart disease and diseases of pulmonary circulation | I26-I28 |
| LC-37 | Nonrheumatic valve disorders | I34-I38 |
| LC-38 | Cardiomyopathy | I42 |
| LC-39 | Cardiac arrest | I46 |
| LC-40 | Cardiac arrhythmias | I47-I49 |
| LC-41 | Heart failure and complications and ill-defined heart disease | I50-I51 |
| LC-42 | Cerebrovascular diseases | I60-I69 |
| LC-43 | Atherosclerosis | I70 |
| LC-44 | Aortic aneurysm and dissection | I71 |
| LC-45 | Acute respiratory diseases other than influenza and pneumonia | J00-J06, J20-J22 |
| LC-46 | Influenza and Pneumonia | J10-J18 |
| LC-47 | Chronic lower respiratory diseases | J40-J47 |
| LC-48 | Pulmonary edema and other interstitial pulmonary diseases | J80-J84 |
| LC-49 | Respiratory failure | J96 |
| LC-50 | Appendicitis, hernia and intestinal obstruction | K35-K46, K56 |
| LC-51 | Cirrhosis and other diseases of liver | K70-K76 |
| LC-52 | Diseases of the musculoskeletal system and connective tissue | M00-M99 |
| LC-53 | Diseases of the urinary system | N00-N39 |
| LC-54 | Pregnancy, childbirth and the puerperium | O00-O99 |
| LC-55 | Certain conditions originating in the perinatal period | P00-P96 |
| LC-56 | Congenital malformations, deformations and chromosomal abnormalities | Q00-Q99 |
| LC-57 | Land transport accidents | V01-V89 |
| LC-58 | Accidental falls | W00-W19 |
| LC-59 | Nonintentional firearm discharge | W32-W34 |
| LC-60 | Accidental drowning and submersion | W65-W74 |
| LC-61 | Accidental threats to breathing | W75-W84 |
| LC-62 | Accidental poisoning | X40-X49 |
| LC-63 | Intentional self-harm [suicide] | X60-X84 |
| LC-64 | Assault [homicide] | X85-Y09 |
| LC-65 | Event of undetermined intent | Y10-Y34 |
