# Supplementary material for: Changes in the rankings of leading causes of death in Japan, Korea, and Taiwan from 1998 to 2018: a comparison of three ranking lists
Source: BMC Public Health. 2022 May 10;22:926. doi: 10.1186/s12889-022-13278-7 (PMC9086411; doi:10.1186/s12889-022-13278-7)
Supplement: Supplementary file 6 — Additional file 6. Table S6. List for ranking leading causes of death by the Institute of Health Metrics and Evaluation [file 12889_2022_13278_MOESM6_ESM.docx]

Table S6. List for ranking leading causes of death by the Institute of Health Metrics and Evaluation (N=137)

| Number | **Name of category** | **ICD10** |
| --- | --- | --- |
| A.1.1 | HIV/AIDS | B20-B24.9 |
| A.2.1 | Tuberculosis | A15-A19.9, B90-B90.9, K67.3, K93.0, M49.0, N74.1, P37.0, U84.3 |
| A.2.2 | Lower respiratory infections | A48.1, A70, B97.4-B97.6, J09-J15.8, J16-J16.9, J20-J21.9, P23.0-P23.4, U04-U04.9 |
| A.2.3 | Upper respiratory infections | J00-J02.8, J03-J03.8, J04-J04.2, J05-J05.1, J06.0-J06.8, J36-J36.0 |
| A.2.4 | Otitis media | H70-H70.9 |
| A.3.1 | Diarrheal diseases | A00-A00.9, A02-A02.0, A02.8-A07, A07.2-A07.4, A08-A09.9, R19.7 |
| A.3.2 | Typhoid and paratyphoid | A01.0-A01.4 |
| A.3.3 | Invasive Non-typhoidal Salmonella (iNTS) | A02.1-A02.2 |
|  | Poliomyelitis | A80-A80.9 |
| A.3.5 | Other intestinal infectious diseases | A07.0-A07.1, A07.8-A07.9 |
| A.4.1 | Malaria | B50-B53.8 |
| A.4.16 | Leprosy | A30-A30.9 |
| A.4.2 | Chagas disease | B57-B57.5, K93.1 |
| A.4.3 | Leishmaniasis | B55.0 |
| A.4.4 | African trypanosomiasis | B56-B56.9 |
| A.4.5 | Schistosomiasis | B65-B65.9 |
| A.4.6 | Cysticercosis | B69-B69.9 |
| A.4.7 | Cystic echinococcosis | B67-B67.4, B67.8-B67.9 |
| A.4.11 | Dengue | A90-A91.9 |
| A.4.12 | Yellow fever | A95-A95.9 |
| A.4.13 | Rabies | A82-A82.9 |
| A.4.14 | Intestinal nematode infections | B77-B77.9 |
| A.4.17 | Ebola | A98.4 |
| A.4.18 | Zika virus | U06-U06.9 |
| A.4.20 | Other neglected tropical diseases | A68-A68.9, A69.2-A69.9, A75-A75.9, A77-A79.9, A92-A94.0, A96-A96.9, A98-A98.3, A98.5-A98.8, B33.0-B33.1, B60-B60.8, B67.5-B67.7, B70-B71.9, B74.3-B75, B83-B83.8, P37.1 |
| A.5.1 | Meningitis | A39-A39.9, A87-A87.9, G00.0-G00.8, G03-G03.8 |
| A.5.2 | Encephalitis | A83-A86.4, B94.1, F07.1, G04-G05.8, G21.3 |
| A.5.3 | Diphtheria | A36-A36.9 |
| A.5.4 | Whooping cough | A37-A37.9 |
| A.5.5 | Tetanus | A33-A35.0 |
| A.5.6 | Measles | B05-B05.9 |
| A.5.7 | Varicella and herpes zoster | B01-B02.9, P35.8 |
| A.5.8 | Acute hepatitis | B15-B17.9, B19-B19.9, B94.2, P35.3 |
| A.5.9 | Other unspecified infectious diseases | A20-A28.9, A32-A32.9, A38-A38.9, A48.2, A48.4-A48.5, A65-A65.0, A69-A69.1, A74, A74.8-A74.9, A81-A81.9, A88-A89.9, B00-B00.9, B03-B04, B06-B06.9, B10-B10.8, B25-B27.9, B29.4, B33, B33.3-B33.8, B47-B48.8, B91, B95-B95.5, G14-G14.6, I00, I02, I02.9, I98.1, K67.8, K75.3, K76.3, K77.0, M49.1, M89.6, P35-P35.2, P35.9, P37, P37.2, P37.5-P37.9, U82-U84, U85-U89, Z16-Z16.3 |
| A.6.1 | Maternal disorders | N96, N98-N98.9, O00-O07.9, O09-O16.9, O20-O26.9, O28-O36.9, O40-O48.1, O60-O77.9, O80-O92.7, O96-O98.6, O98.8-O99.9 |
| A.6.2 | Neonatal disorders | P00-P04.2, P04.5-P05.9, P07-P15.9, P19-P22.9, P24-P29.9, P36-P36.9, P38-P39.9, P50-P61.9, P70-P70.1, P70.3-P72.9, P74-P78.9, P80-P81.9, P83-P84, P90-P94.9, P96, P96.3-P96.4, P96.8 |
| A.7.1 | Protein-energy malnutrition | E40-E46.9, E64.0 |
| A.7.2 | Iodine deficiency | E00-E02.8 |
| A.7.4 | Iron-deficiency anemia | D50.1-D50.8 |
| A.7.5 | Other nutritional deficiencies | D51-D52.0, D52.8-D53.9, E00-E02, E51-E61.9, E63-E64, E64.2-E64.9, M12.1 |
| B.1.1 | Lip and oral cavity cancer | C00-C08.9, D10.0-D10.5, D11-D11.9 |
| B.1.2 | Nasopharynx cancer | C11-C11.9, D10.6 |
| B.1.3 | Other pharynx cancer | C09-C10.9, C12-C13.9, D10.7 |
| B.1.4 | Esophageal cancer | C15-C15.9, D00.1, D13.0 |
| B.1.5 | Stomach cancer | C16-C16.9, D00.2, D13.1, D37.1 |
| B.1.6 | Colon and rectum cancer | C18-C21.9, D01.0-D01.3, D12-D12.9, D37.3-D37.5 |
| B.1.7 | Liver cancer | C22-C22.9, D13.4 |
| B.1.8 | Gallbladder and biliary tract cancer | C23-C24.9, D13.5 |
| B.1.9 | Pancreatic cancer | C25-C25.9, D13.6-D13.7 |
| B.1.10 | Larynx cancer | C32-C32.9, D02.0, D14.1, D38.0 |
| B.1.11 | Tracheal, bronchus, and lung cancer | C33-C34.9, D02.1-D02.3, D14.2-D14.3, D38.1 |
| B.1.12 | Malignant skin melanoma | C43-C43.9, D03-D03.9, D22-D23.9, D48.5 |
| B.1.13 | Non-melanoma skin cancer | C44-C44.9, D04-D04.9, D49.2 |
| B.1.14 | Breast cancer | C50-C50.9, D05-D05.9, D24-D24.9, D48.6, D49.3 |
| B.1.15 | Cervical cancer | C53-C53.9, D06-D06.9, D26.0 |
| B.1.16 | Uterine cancer | C54-C54.9, D07.0-D07.2, D26.1-D26.9 |
| B.1.17 | Ovarian cancer | C56-C56.9, D27-D27.9, D39.1 |
| B.1.18 | Prostate cancer | C61-C61.9, D07.5, D29.1, D40.0 |
| B.1.19 | Testicular cancer | C62-C62.9, D29.2-D29.8, D40.1-D40.8 |
| B.1.20 | Kidney cancer | C64-C65.9, D30.0-D30.1, D41.0-D41.1 |
| B.1.21 | Bladder cancer | C67-C67.9, D09.0, D30.3, D41.4-D41.8, D49.4 |
| B.1.22 | Brain and nervous system cancer | C70-C72.9 |
| B.1.23 | Thyroid cancer | C73-C73.9, D09.3, D09.8, D34-D34.9, D44.0 |
| B.1.24 | Mesothelioma | C45-C45.9 |
| B.1.25 | Hodgkin lymphoma | C81-C81.9 |
| B.1.26 | Non-Hodgkin lymphoma | C82-C86.6, C96-C96.9 |
| B.1.27 | Multiple myeloma | C88-C90.9 |
| B.1.28 | Leukemia | C91-C95.9 |
| B.1.29 | Other malignant cancers | C17-C17.9, C30-C31.9, C37-C38.8, C40-C41.9, C47-C4A, C51-C52.9, C57-C57.8, C58-C58.0, C60-C60.9, C63-C63.8, C66-C66.9, C68.0-C68.8, C69-C69.9, C74-C75.8, D07.4, D09.2, D13.2-D13.3, D14.0, D15-D16.9, D28.0-D28.1, D28.7, D29.0, D30.2, D30.4-D30.8, D31-D31.9, D35-D35.2, D35.5-D36, D36.1-D36.7, D37.2, D38.2-D38.5, D39.2, D39.8, D41.2-D41.3, D44.1-D44.8, D48.0-D48.4 |
| B.1.30 | Other neoplasms | D32-D33.9, D35.3-D35.4, D42-D43.9, D45-D47.9, D49.6, K62.0-K62.1, K63.5, N60-N60.9, N84.0-N84.1, N87-N87.9 |
| B.2.1 | Rheumatic heart disease | I01-I01.9, I02.0, I05-I09.9 |
| B.2.2 | Ischemic heart disease | I20-I25.9 |
| B.2.3 | Stroke | G45-G46.8, I60-I63.9, I65-I66.9, I67.0-I67.3, I67.5-I67.6, I68.1-I68.2, I69.0-I69.3 |
| B.2.4 | Hypertensive heart disease | I11-I11.9 |
| B.2.5 | Non-rheumatic valvular heart disease | I34-I37.8 |
| B.2.6 | Cardiomyopathy and myocarditis | B33.2, I40-I41.9, I42.1-I42.8, I43-I43.9, I51.4 |
| B.2.7 | Atrial fibrillation and flutter | I48-I48.9 |
| B.2.8 | Aortic aneurysm | I71-I71.9 |
| B.2.9 | Peripheral artery disease | I70.2-I70.8, I73-I73.9 |
| B.2.10 | Endocarditis | I33-I33.9, I38-I39.9 |
| B.2.11 | Other cardiovascular and circulatory diseases | I28-I28.8, I30-I31.1, I31.8-I32.8, I47-I47.9, I51.0-I51.3, I68.0, I72-I72.9, I77-I83.9, I86-I89.0, I89.9, I98, K75.1 |
| B.3.1 | Chronic obstructive pulmonary disease | J41-J44.9 |
| B.3.2 | Pneumoconiosis | J60-J63.8, J65-J65.0, J92.0 |
| B.3.3 | Asthma | J45-J46.9 |
| B.3.4 | Interstitial lung disease and pulmonary sarcoidosis | D86-D86.2, D86.9, J84-J84.9 |
| B.3.5 | Other chronic respiratory diseases | G47.3, J30-J35.9, J37-J39.9, J66-J68.9, J70, J70.8-J70.9, J82, J91-J92, J92.9 |
| B.4.1 | Cirrhosis and other chronic liver diseases | B18-B18.9, I85-I85.9, I98.2, K70-K70.3, K71.7, K74-K74.9, K75.2, K75.4-K76.2, K76.4-K76.9, K77.8 |
| B.4.2 | Upper digestive system diseases | K21-K21.9, K22.7, K25-K29.9, K31, K31.1-K31.6, K31.8 |
| B.4.3 | Appendicitis | K35-K37.9, K38.3-K38.9 |
| B.4.4 | Paralytic ileus and intestinal obstruction | K56-K56.9 |
| B.4.5 | Inguinal, femoral, and abdominal hernia | K40-K42.9, K44-K46.9 |
| B.4.6 | Inflammatory bowel disease | K50-K52.9, M09.1 |
| B.4.7 | Vascular intestinal disorders | K55-K55.9 |
| B.4.8 | Gallbladder and biliary diseases | K80-K83.9 |
| B.4.9 | Pancreatitis | K85-K86.9 |
| B.4.10 | Other digestive diseases | I84-I84.9, K20-K20.9, K22-K22.6, K22.8-K24, K31.0, K31.7, K38-K38.2, K57-K62, K62.2-K62.6, K62.8-K62.9, K64-K64.9, K66.8, K67, K68-K68.9, K77, K90-K90.9, K92.8, K93.8 |
| B.5.1 | Alzheimer disease and other dementias | F00-F03.9, G30-G31.1, G31.8-G31.9 |
| B.5.2 | Parkinson disease | G20-G20.9 |
| B.5.3 | Epilepsy | G40-G41.9 |
| B.5.4 | Multiple sclerosis | G35-G35.9 |
| B.5.5 | Motor neuron disease | G12.2-G12.9 |
| B.5.7 | Other neurological disorders | G10-G12.1, G13-G13.8, G23-G24, G24.1-G25.0, G25.2-G25.3, G25.5, G25.8-G26.0, G36-G37.9, G61-G61.9, G70-G72, G72.2-G73.7, G90-G90.9, G95-G95.9, M33-M33.9 |
| B.6.5 | Eating disorders | F50.0-F50.5 |
| B.7.1 | Alcohol use disorders | F10-F10.9, G31.2, G72.1, P04.3, Q86.0, R78.0, X45-X45.9, X65-X65.9, Y15-Y15.9 |
| B.7.2 | Drug use disorders | F11-F16.9, F18-F19.9, P04.4, P96.1, R78.1-R78.5 |
| B.8.1 | Diabetes mellitus | E10-E10.1, E10.3-E11.1, E11.3-E11.9, P70.2 |
| B.8.2 | Chronic kidney disease | D63.1, E10.2, E11.2, I12-I13.9, N02-N08.8, N15.0, N18-N18.9, Q61-Q62.8 |
| B.8.3 | Acute glomerulonephritis | N00-N01.9 |
| B.9.3 | Bacterial skin diseases | A46-A46.0, A66-A67.9, I89.1-I89.8, L00-L05.9, L08-L08.9, L88, L97-L98.4, M72.5-M72.6 |
| B.9.11 | Decubitus ulcer | L89-L89.9 |
| B.9.12 | Other skin and subcutaneous diseases | D86.3, L10-L14.0, L51-L51.9 |
| B.11.1 | Rheumatoid arthritis | M05-M06.9, M08.0-M08.8 |
| B.11.6 | Other musculoskeletal disorders | I27.1, I67.7, L93-L93.2, M00-M03.0, M03.2-M03.6, M07-M08, M08.9-M09.0, M09.2-M09.8, M30-M32.9, M34-M36.8, M40-M43.1, M65-M65.0, M71.0-M71.1, M80-M82.8, M86.3-M86.4, M87-M87.0, M88-M89.0, M89.5, M89.7-M89.9 |
| B.12.1 | Congenital birth defects | P96.0, Q00-Q07.9, Q10.4-Q18.9, Q20-Q28.9, Q30-Q36, Q37-Q45.9, Q50-Q60.6, Q63-Q86, Q86.1-Q87.8, Q89-Q89.8, Q90-Q93.9, Q95-Q99.8 |
| B.12.2 | Urinary diseases and male infertility | N10-N12.9, N15, N15.1-N16.8, N20-N23.0, N25-N28.1, N29-N32.0, N32.3-N32.4, N34-N34.3, N36-N36.9, N39-N39.2, N41-N41.9, N44-N44.0, N45-N45.9, N49-N49.9 |
| B.12.3 | Gynecological diseases | D25-D26, D28.2, E28.2, N72-N72.0, N75-N77.8, N80-N81.9, N83-N83.9 |
| B.12.4 | Hemoglobinopathies and hemolytic anemias | D55-D58.9, D59.1, D59.3, D59.5, D60-D61.9, D64.0 |
| B.12.5 | Endocrine, metabolic, blood, and immune disorders | D52.1, D59.0, D59.2, D59.6, D66-D67, D68.0-D69.8, D70-D75.8, D76-D78.8, D86.8, D89-D89.3, E03-E07.1, E09-E09.9, E15.0, E16.0-E16.9, E20-E28.1, E28.3-E34.8, E36-E36.8, E65-E68, E70-E85.2, E88-E89.9, G24.0, G25.1, G25.4, G25.6-G25.7, G72.0, G93.7, G97-G97.9, I95.2-I95.3, I97-I97.9, I98.9, J70.0-J70.5, J95-J95.9, K43-K43.9, K62.7, K91-K91.9, K94-K95.8, M87.1, N14-N14.4, N65-N65.1, N99-N99.9, P96.2, P96.5, R50.2 |
| B.12.7 | Sudden infant death syndrome | R95-R95.9 |
| C.1.1 | Road injuries | V01-V04.9, V06-V80.9, V82-V82.9, V87.2-V87.3 |
| C.1.2 | Other transport injuries | V00-V00.8, V05-V05.9, V81-V81.9, V83-V86.9, V88.2-V88.3, V90-V98.8 |
| C.2.1 | Falls | W00-W19.9 |
| C.2.2 | Drowning | W65-W70.9, W73-W74.9 |
| C.2.3 | Fire, heat, and hot substances | X00-X06.9, X08-X19.9 |
| C.2.4 | Poisonings | X46-X48.9 |
| C.2.5 | Exposure to mechanical forces | W20-W38.9, W40-W43.9, W45.0-W45.2, W46-W46.2, W49-W52 |
| C.2.6 | Adverse effects of medical treatment | Y40-Y84.9, Y88-Y88.3 |
| C.2.7 | Animal contact | W52.0-W62.9, W64-W64.9, X20-X29.9 |
| C.2.8 | Foreign body | W44-W45, W45.3-W45.9, W75-W75.9, W78-W80.9, W83-W84.9 |
| C.2.9 | Environmental heat and cold exposure | L55-L55.9, L56.3, L56.8-L56.9, L58-L58.9, W88-W94.9, W97.9, W99-W99.9, X30-X32.9, X39-X39.9 |
| C.2.10 | Exposure to forces of nature | X33-X38.9 |
| C.2.11 | Other unintentional injuries | W39-W39.9, W77-W77.9, W81-W81.9, W85-W87.9, X50-X54.9, X57-X58.9 |
| C.3.1 | Self-harm | X60-X64.9, X66-X84.9, Y87.0 |
| C.3.2 | Interpersonal violence | X85-Y08.9, Y87.1 |
| C.3.3 | Conflict and terrorism | U00-U03, Y36-Y38.9, Y89.1 |
| C.3.4 | Executions and police conflict | Y35-Y35.9, Y89.0 |
